# Treatment of Porphyria Cutanea Tarda Scarring With Combination Laser Treatment and a Pilot Use of Artificial Intelligence to Quantify Laser Results

**DOI:** 10.1111/jocd.70056

**Published:** 2025-04-01

**Authors:** Chelsea E. Kesty, Katarina R. Kesty

**Affiliations:** ^1^ St. Petersburg Skin and Laser St. Petersburg Florida USA; ^2^ Kesty AI St. Petersburg Florida USA

**Keywords:** aesthetic medicine, laser resurfacing, medical cosmetology, medical therapy, skin resurfacing, vascular

## Abstract

**Background:**

Porphyria cutanea tarda (PCT) is the most common subtype of porphyria and results from a deficiency of the enzyme uroporphyrinogen decarboxylase. Even after successful treatment, patients can be left with significant scarring, and there is little published data on the safety and efficacy of light‐based or laser‐based therapies.

**Methods:**

This report examines a case of a 47‐year‐old male with PCT secondary to HCV, treated with a combination of fractionated erbium‐doped yttrium‐aluminum‐garnet (Er:YAG), intense pulsed light (IPL), and carbon dioxide (CO_2_) lasers to address significant scarring and residual skin damage. An artificial intelligence model was used to quantify the results of the laser procedures.

**Results:**

After combination laser treatment, the patient exhibited marked improvements in skin texture, reduction in scar visibility, and diminished hyperpigmentation. The artificial intelligence algorithm quantified the laser results and showed improvements in the scores used in the large language model.

**Conclusion:**

In this patient, customizing a combination of lasers to target different layers of the skin to achieve comprehensive improvement: erbium primarily addressed superficial irregularities and pigmentation, while CO_2_ promoted deeper collagen remodeling. The use of artificial intelligence to quantify the positive results in this case is in line with the clinical evaluations and photos.

**Abbreviations**
CO_2_
: carbon dioxide
Er‐YAG: erbium‐doped yttrium‐aluminum‐garnet
HCV: hepatitis C virus
IPL: intense pulsed light
PCT: porphyria cutanea tarda

## 1 Background

Porphyria cutanea tarda (PCT) is the most common subtype of porphyria and results from a deficiency of the enzyme uroporphyrinogen decarboxylase [1, 2]. This condition typically manifests as blistering skin lesions, photosensitivity, hyperpigmentation, and scarring, especially on sun‐exposed areas. PCT is often associated with hepatic dysfunction, particularly hepatitis C virus (HCV) infection, which can exacerbate symptoms by increasing hepatic iron levels and other liver abnormalities. Even after successful treatment, patients can be left with significant scarring, and there is little published data on the safety and efficacy of light‐based or laser‐based therapies. This report examines a case of a 47‐year‐old male with PCT secondary to HCV, treated with a combination of fractionated Erbium‐doped yttrium‐aluminum‐garnet (Er:YAG), intense pulsed light (IPL) and carbon dioxide (CO_2_) lasers to address significant scarring and residual skin damage. The artificial intelligence (AI) algorithm utilized in this study was developed by Kesty AI (Kesty AI, Florida, USA) to analyze patient photographs and predict scores on various scales of patient characteristics [3]. Patient images were uploaded to the Kesty AI platform (www.kesty.ai), where they were processed by the proprietary algorithm. The machine learning model generated ratings for each photograph based on both scales.

This algorithm was trained using a comprehensive dataset of thousands of patient images, all of which were evaluated by a board‐certified dermatologist. The corresponding ratings were employed to ‘train’ the machine learning model, which was subsequently subjected to rigorous validation to ensure its accuracy in predicting patient characteristics from facial photographs. Among the attributes the model can assess are the Fitzpatrick Wrinkle Scale, Glogau Wrinkle Scale, Kesty Redness Scale and the Kesty Hyperpigmentation Scale, all of which were integral to this study. Machine learning algorithms are favored over manual evaluations in research due to their ability to standardize results, thereby minimizing the variability and potential for human error in assessing outcomes, such as the effects of laser treatments or other cosmetic procedures. This standardization is crucial in ensuring consistent and reliable evaluations of before‐and‐after results.

## 2 Objective

**FIGURE 1:**
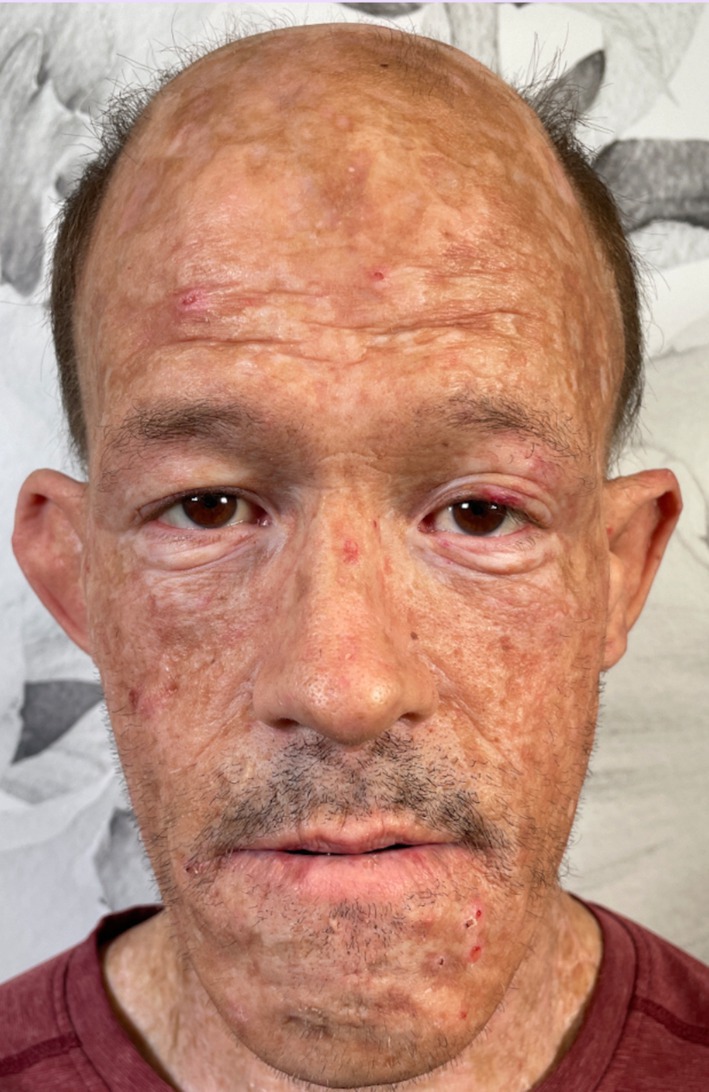
Patient with scarring due to porphyria cutanea tarda.

A 47‐year‐old Caucasian male presented with a history of recurrent blistering and erosions on sun‐exposed areas, predominantly on his face, scalp, forearms, and hands (Figure 1). The patient had a medical history significant for PCT secondary to hepatitis C infection, which he acquired through a needle stick injury. The patient had completed treatment 2 years prior to presentation at our Laser Clinic. However, despite resolving his active lesions, the patient had significant residual scarring and hyperpigmentation, which caused social discomfort and functional limitations in his daily activities. The patient reported difficulty with facial movements, including making facial expressions, opening his mouth, and moving his head side to side and up and down. Limited movement of facial and neck muscles was appreciated on physical exam at the time of presentation. After evaluating treatment options, the patient was scheduled for a series of combination laser treatments using advanced technologies of IPL, Er:YAG, and CO_2_ lasers, targeted at improving skin texture, reducing scarring, and promoting collagen remodeling. The treatment plan involved six sessions spaced 4–6 weeks apart. At sessions 1, 2, and 5, a customized combination of IPL and fractionated Er:YAG laser at 2940 nm was utilized. The patient's face, neck, and scalp were treated. The IPL 560 filter was utilized with energy ranging between 9–11 J/cm^2^ and pulse width of 20–25 ms. Following that, the IPL 515 filter was utilized with energy ranging between 6–10 J/cm^2^ and pulse width of 20–25 ms. The Er:YAG settings ranged between 150 and 300 μm depth and 5.5%–11% densities. At sessions 3, 4, and 6, a fractionated CO_2_ laser at 10 600 nm was utilized for treatment of face, neck, and scalp. Two passes were done on the patient's face with parameters of 5% density and 12.5 mJ energy per pulse with a 10‐mm spot size, followed by 50 mJ energy, 40% density, and 6 mm spot size. The scalp and chest were treated with 5% density and 12.5 mJ energy per pulse with a 10‐mm spot size. There was no photosensitivity or increased response to the lasers, including IPL, appreciated after laser sessions in this case. The patient's clinical photographs were run through an artificial intelligence large language model to evaluate for redness, hyperpigmentation, Glogau wrinkle scale, and Fitzpatrick wrinkle scale both before and after laser treatment (Kesty AI, St. Petersburg, FL).

**FIGURE 2:**
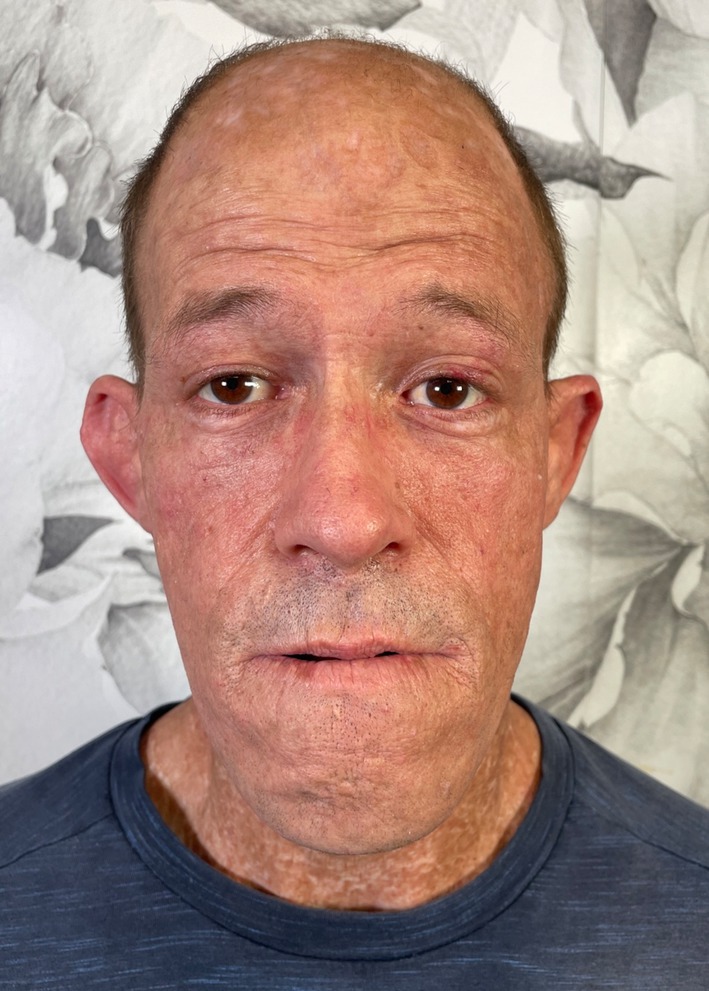
Patient with scarring due to porphyria cutanea tarda after a combination of laser treatments.

The patient tolerated the procedures well, and no post‐operative adverse events were reported. The patient tolerated all laser sessions without significant complications. He reported mild discomfort, erythema, and transient edema after each session, which resolved within 5 days. No recurrence of blistering or further exacerbation of scarring was observed. After the final session, the patient exhibited marked improvements in skin texture, reduction in scar visibility, and diminished hyperpigmentation (Figure 2). He expressed satisfaction with the aesthetic outcomes and reported increased confidence in social settings. Photographic documentation showed smoother skin texture, reduced discoloration, and decreased depth of scars on treated areas (Figure 2). The artificial intelligence model that was used to evaluate the patient photographs computed a 2 on the Kesty Hyperpigmentation Scale for the before and after photographs. For the Kesty Redness scale, the artificial intelligence model computed a 2 for the before photographs and a 0 on the scale for the after. The computed Glogau wrinkle scale was a 4 for both the before and after photographs. The Fitzpatrick Wrinkle Scale that was assigned to the patient photos for before and after treatment by the artificial intelligence model was a 9 for the before and a 7 for the after photos. The use of artificial intelligence to quantify the positive results in this case is in line with the clinical evaluations and photos.

## 3 Discussion

PCT presents unique therapeutic challenges due to its chronicity, susceptibility to recurrent lesions, and the cosmetic burden of resulting scars and pigmentation changes. Laser therapy, particularly using a custom combination of fractionated lasers, has shown promising results in improving scar quality and pigmentation issues by stimulating dermal remodeling and melanocyte redistribution [4, 5]. Fractionated erbium and CO_2_ lasers are commonly used for scar revision due to their dual benefits of ablative and non‐ablative effects, which promote both immediate resurfacing and gradual collagen regeneration [6]. In this patient, customizing a combination of lasers to target different layers of the skin to achieve comprehensive improvement: erbium primarily addressed superficial irregularities and pigmentation, while CO_2_ promoted deeper collagen remodeling. There is a concern for photosensitivity or an exaggerated reaction to laser and other light‐based devices with PCT; however, this patient tolerated all laser sessions well and no aberrant reaction to the lasers, in particular the IPL, was appreciated. This may be due to the timeline of the PCT, as it had been treated several years ago and the patient was no longer having any symptoms of PCT.

The use of artificial intelligence to quantify the outcome of this laser case may be expanded to further laser trials and cases in order to standardize results. This case highlights the successful use of a custom sequence of laser technologies including IPL, fractionated erbium, and CO_2_ lasers to manage scarring and pigmentation in a patient with PCT secondary to HCV. The unique challenges of PCT patients include the risk of phototoxicity and increased sensitivity, especially with light‐based therapy. Adhering to conservative parameters and post‐treatment care was crucial to minimize risks, as this patient's protocol demonstrates. It is imperative for optimizing patient outcomes that laser surgeons have access and familiarity with a variety of lasers to create unique laser sequences to address the variety of skin concerns every patient presents with. The patient's cosmetic outcomes and quality of life improvement provide evidence of an effective approach for residual skin damage in PCT cases.

## Author Contributions

K.R.K. and C.E.K. conceived the study, developed the laser protocols and settings, wrote and revised the manuscript, and funded the study. All authors have reviewed and approved the article for submission.

## Conflicts of Interest

K.R.K. is the Founder of Kesty AI, which was used in this paper.

## Data Availability

The data that support the findings of this study are available on request from the corresponding author. The data are not publicly available due to privacy or ethical restrictions.
